# Pulmonary co-infections by *Pneumocystis jirovecii* and *Herpesviridae*: a seven-year retrospective study

**DOI:** 10.1186/s12941-023-00663-2

**Published:** 2024-01-20

**Authors:** Alan Rucar, Anne Totet, Yohann Le Govic, Baptiste Demey, Céline Damiani

**Affiliations:** 1 Laboratoire de Parasitologie et Mycologie Médicales, Centre de Biologie Humaine, CHU Amiens-Picardie, 1 rond-point du Pr Cabrol, 80054 Amiens Cedex 1, France; 2 Agents Infectieux, Résistance et Chimiothérapie (AGIR), UR 4294, Université de Picardie Jules Verne, 1 rue des Louvels, 80037 Amiens Cedex 1, France; 3 Laboratoire de Virologie, Centre de Biologie Humaine, CHU Amiens-Picardie, 1 rond-point du Pr Cabrol, 80054 Amiens Cedex 1, France

**Keywords:** *Pneumocystis jirovecii*, *Pneumocystis* pneumonia, *Pneumocystis* pulmonary colonization, Pulmonary coinfections, *Herpesviridae*

## Abstract

**Background:**

*Pneumocystis jirovecii (P. jirovecii)* is an opportunistic fungus responsible for *Pneumocystis* pneumonia (PCP) in deeply immunocompromised patients and for pulmonary colonization in individuals with mild immunosuppression or impaired respiratory function. PCP and Cytomegalovirus (CMV) co-infections have been widely described whereas those involving other Herpesviruses (HVs) such as Epstein-Barr virus (EBV), Herpes simplex virus type 1 and type 2 (HSV-1 and  -2), and Varicella zoster virus (VZV) remain scarce. To date, no data are available concerning HVs co-infections in *P. jirovecii* colonization.

**Methods:**

Our main objective was to evaluate the frequency of HVs in bronchoalveolar lavage fluid (BALF) samples from patients with PCP or with pulmonary colonization. The secondary objective was to assess the relationship between HVs and the mortality rate in PCP patients. A retrospective single-center study over a seven-year period was conducted. All patients with *P. jirovecii* detected using PCR in a BALF sample and for whom a PCR assay for HVs detection was performed were included in the study.

**Results:**

One hundred and twenty-five patients were included, corresponding to 77 patients with PCP and 48 colonized patients. At least one HV was detected in 54/77 (70.1%) PCP patients and in 28/48 (58.3%) colonized patients. EBV was the most frequent in both groups. Furthermore, the 30-day survival rate in PCP patients was significantly lower with [EBV + CMV] co-infection than that with EBV co-infection, [EBV + HSV-1] co-infection and without HV co-infection.

**Conclusion:**

Our results show that the frequency of HV, alone or in combination is similar in PCP and colonization. They also suggest that [EBV + CMV] detection in BALF samples from PCP patients is associated with an increased mortality rate, underlying the significance to detect HVs in the course of PCP.

## Introduction

*Pneumocystis jirovecii* (*P. jirovecii*) is an opportunistic fungus responsible for *Pneumocystis* pneumonia (PCP) in deeply immunocompromised patients. Spontaneous evolution of PCP is fatal and this invasive fungal disease was a major cause of morbidity and mortality among HIV-infected people during the 80 and 90 s [1]. Nowadays, patients with hematological malignancy (HM), solid-organ tumor, solid-organ transplantation, primary immune deficiency and/or receiving long-term (> 3 months) or high-dose (> 0.5 mg/kg/day) corticosteroids or other immunosuppressive drugs present an increased risk of developing PCP [2]. Thus, the at-risk population is now clearly recognized and PCP incidence in immunocompromised HIV-uninfected patients with no prophylaxis is estimated between 5 and 15% [3]. *Pneumocystis* detection in individuals with mild immunodeficiency or impaired respiratory function may also reflect pulmonary colonization. *Pneumocystis* colonization has been described in patients with chronic lung diseases such as chronic obstructive pulmonary disease but also occurs in immunosuppressed population [4]. Meanwhile, *Herpesviridae*/Herpesviruses (HVs), such as Cytomegalovirus (CMV), Epstein-Barr virus (EBV), Herpes simplex viruses type 1 and type 2 (HSV-1 and  -2) or Varicella zoster virus (VZV) can also be responsible for severe pneumonia, especially in critically ill patients [5]. Moreover, rising numbers of critically ill patients are henceforth immunocompromised and account for approximately 30% of all ICU admissions [6]. Pulmonary co-infections with *P. jirovecii* and CMV have widely been described [7–13], whereas those relating the combination of the fungus with other HVs are still scarce [14, 15]. Moreover the combination of *P. jirovecii* with CMV and/or HSV has been associated with a poor prognosis, especially in HIV-uninfected patients. Indeed, Fillatre et al. investigated the prognosis of severe PCP in 70 HIV-negative patients admitted to ICU for acute respiratory failure and showed that in-ICU mortality was associated with the detection of HSV or CMV on bronchoalveolar lavage fluid (BALF) samples [14]. Likewise, Korkmaz Ekren et al. compared morbidity and mortality stratified by CMV co-infection status in 43 PCP patients and found that 30-day mortality was significantly lower in the group with PCP alone as compared to the CMV co-infection group [11]. In addition, these reports were related to PCP cases and no data regarding HVs in *Pneumocystis* pulmonary colonization is currently available.

In this context, our main objective was to evaluate the frequency of HVs (CMV, EBV, HSV-1 and  -2, and VZV), alone or in combination, in BALF samples from patients with PCP or pulmonary colonization. The secondary objective was to assess the mortality rate according to the presence of HVs in PCP patients.

## Patients and methods

### Patients

This retrospective study was conducted in Amiens-Picardy University Hospital. From May 2013 to May 2020, patients who were PCR-positive for *P. jirovecii* and who were tested for five herpesviruses (CMV, EBV, HSV-1 and  -2, VZV) by PCR assay were included in the study. *P. jirovecii* and HVs detections were performed either in a single BALF sample or in two BALF samples collected less than 2 days apart with *P. jirovecii* detection in one of the two samples and HV detection in the other. All patients were followed-up for at least one year.

### Data collection

For each patient, the following data were collected: age, gender, underlying disease, comorbidity factors, immunosuppressive regimens, PCP regimen, anti-viral therapy, and patient outcome including 30-day mortality.

Laboratory findings regarding *P. jirovecii* detection were collected. Microscopic examination was performed with methanol-Giemsa staining combined with an immunofluorescence assay (Monofluokit *Pneumocystis*; Bio-Rad, Marnes-La-Coquette,France) during the first 5 years of the study and thereafter with a stilbene-derived fluorescent dye (Uvibio^®^; LDBIO Diagnostics, Lyon, France). *P. jirovecii* DNA was also detected using an in-house quantitative PCR (qPCR) assay targeting the mitochondrial large subunit rRNA gene as previously described [16].

For each patient, results of HVs DNA detection were compiled. PCR assays were performed with Argene^®^ (bioMérieux) kits with Applied Biosystems 7300^®^ system during the first four years of the study and thereafter with Altona Diagnostics^®^ kits with the m2000 RealTime System (ABBOTT^®^). In 2017, an intra-laboratory method comparison had shown good agreement between these two assays. The characteristics of the virus PCR kits used over the study period are summarized in Table 1.

**Table 1:** Virus PCR kits used between 2013 and 2020

	Bronchoalveolar lavage fluid samples		Blood samples
**CMV**	CMV R-gene^®^ Biomérieux, France	RealStar^®^ CMV PCR Kit 1.0 Altona diagnostics, Germany	Abbott Real Time CMV^®^ Abbott molecular inc, USA
**EBV**	EBV R-gene^®^ Biomérieux, France	RealStar^®^ EBV PCR Kit 1.0 Altona diagnostics, Germany	Abbott Real Time EBV^®^ Abbott molecular inc, USA
**HSV1/2 and VZV**	HSV1 HSV2 VZV R-gene^®^ Biomérieux, France	RealStar^®^*alpha* Herpesvirus PCR Kit 1.0 Altona diagnostics, Germany	/

CMV, Cytomegalovirus; EBV, Epstein-Barr virus; HSV1/2, Herpes simplex viruses type 1 and type 2; VZV, Varicella zoster virus

### Definitions

The diagnosis of proven PCP or possible PCP was determined by criteria used in our lab. Proven PCP was defined as a positive microscopic detection of *P. jirovecii* regardless of the staining used for the detection and/or a fungal load higher than 10^7^ DNA copies/ml in BALF samples. Possible PCP was defined as a negative microscopic detection and a fungal load below 10^7^ DNA copies/ml in patients who received a specific anti-*Pneumocystis* treatment. The diagnosis of colonization was then established with criteria used in our lab and adapted from previously published data [16]. Colonization was defined as a negative microscopic detection, a fungal load below 10^7^ copies/ml in BALF sample and a clinical improvement without any anti-*Pneumocystis* treatment. Patients were assigned to the PCP group (proven or possible) or the colonization group after review of the clinical and biological records.

### Statistical analysis

Statistical analyses were performed using R-project^®^ version 4.0.3 and Graphpad prism version 8.0.2. Chi-squared test and Fisher’s exact test were used to compare qualitative variables. Quantitative variables with Gaussian distribution were analyzed using Student’s test and ANOVA test. Results were expressed as means with standard deviation (SD). A p value < 0.05 was considered significant. Survival curves were calculated according to Kaplan-Meier method and compared with the log-rank test.

## Results

### Patient characteristics

One hundred and twenty-five patients met the inclusion criteria with all data available for the analysis. Of these, 77 presented with proven or possible PCP and 48 were colonized by the fungus. Demographic and clinical characteristics are presented in Table 2.

**Table 2:** Demographic and clinical characteristics of the 125 patients included in the study

	PCP (n = 77)	*P. jirovecii* pulmonary colonization (n = 48)	*P* value
Mean age ± SD (years)	59.5 +/- 14	62.7 +/- 12.8	0.19^*^
Male sex, n (%)	50 (64.9)	31 (64.6)	0.96^**^
Underlying disease, n (%)			
Hematological malignancy	37 (48)	19 (39.6)	0.35^**^
Solid organ or hematopoietic stem cell transplant	26 (33.7)	13 (27.1)	0.43^**^
Solid tumor	14 (18.2)	7 (14.6)	0.60^**^
Autoimmune disease	9 (11.7)	5 (10.4)	0.83^**^
Lung disease	7 (9.1)	18 (37.5)	**< 0.01** ^**^
HIV infection	5 (6.5)	0 (0)	0.07^**^
Comorbidity, n (%)			
Cardiovascular disease	32 (41.5)	28 (58.3)	0.07^**^
Diabetes	13 (16.8)	6 (12.5)	0.50^**^
Renal failure	9 (11.7)	2 (4.2)	0.14^**^
Immunosuppressive treatment, n (%)			
Corticosteroids	22 (28.6)	17 (35.4)	0.44^**^
Cytotoxic chemotherapy	23 (29.9)	12 (25)	0.52^**^
Anti-*Pneumocystis* treatment, n (%)	77 (100)	0 (0)	**< 0.01** ^**^
Antiviral treatment, n (%)	24 (31.2)	6 (12.5)	**0.017** ^**^

Bold font indicates statistical significance

SD, standard deviation; PCP, *Pneumocystis pneumonia*

^*^ Student’s test

^**^ Chi-squared test

In the PCP group, the mean age was 59.5 (+/- 14) years old and the sex ratio was 1.77. Underlying conditions consisted in HM (n = 37, 48%), solid organ or hematopoietic stem cell (HSC) transplantation (n = 26, 33.7%), solid tumor (n = 14, 18.2%), autoimmune disease (n = 9, 11.7%), lung disease (n = 7, 9.1%) and HIV infection (n = 5, 6.5%). Comorbidities were cardiovascular disease (n = 32, 41.5%), diabetes (n = 13, 16.8%) and renal failure (n = 9, 11.7%). At time of PCP diagnosis, 22 (28.6%) and 23 (29.9%) patients received corticosteroids and cytotoxic chemotherapy, respectively. Anti-*Pneumocystis* treatment was administrated to all patients and antiviral treatment was needed in 24/77 (31.2%) of them.

In the colonization group, the mean age was 62.7 (+/- 12.8) years old and the sex ratio was 1.82. Underlying conditions consisted in HM (n = 19, 39.6%), solid organ or HSC transplantation (n = 13, 27.1%), solid tumor (n = 7, 14.6%), autoimmune disease (n = 5, 10.4%), lung disease (n = 18, 37.5%). Comorbidities were cardiovascular disease (n = 28, 58.3%), diabetes (n = 6, 12.5%) and renal failure (n = 2, 4.2%). At time of colonization diagnosis, 17 (35.4%) and 12 (25%) patients received corticosteroids and cytotoxic chemotherapy, respectively. No patient received anti-*Pneumocystis* treatment while antiviral treatment was needed for 6 (12.5%) of them.

There was a significant difference between the PCP group and the colonization group regarding the presence of an underlying lung disease, and the use of anti-*Pneumocystis* and antiviral treatments (p < 0.01, p < 0.01 and p = 0.017, respectively).

### Distribution of HVs according to the clinical form of *Pneumocystis* infection

**Fig. 1:**
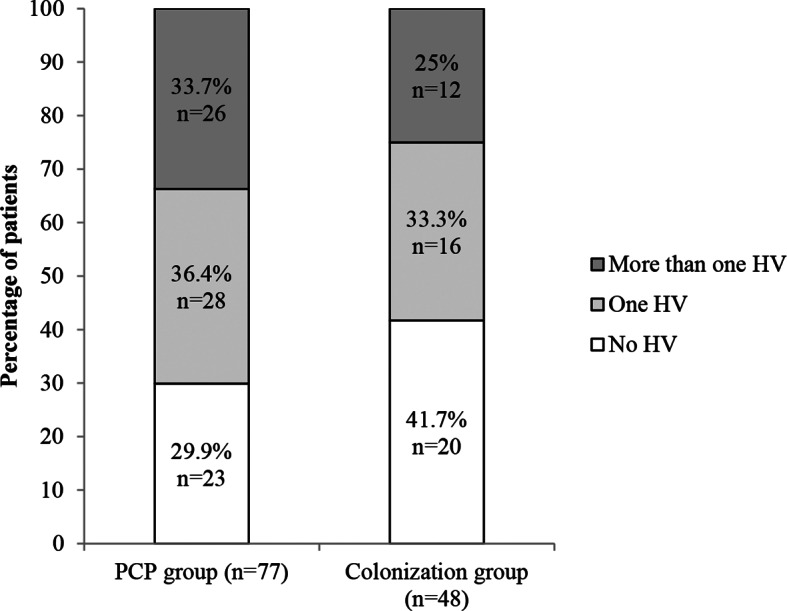
Results of *Herpesviridae* detection in bronchoalveolar lavage fluid sample according to *Pneumocystis* infection. HV, *Herpesviridae*; PCP, *Pneumocystis* pneumonia

In the PCP group, HVs were detected in 54/77 patients (70.1%); a single virus was detected in 28/77 (36.4%) patients while the remaining 26/77 (33.7%) patients harbored more than one virus. In the colonization group, at least one HV was detected in 28/48 patients (58.3%). A single virus was detected in 16/48 (33.3%) patients while the remaining 12/48 (25%) patients harbored more than one virus (Fig. 1).

There was no significant difference between the two groups of patients regarding HVs detection. Demographic and clinical parameters were also classified according to HVs PCR results (Tables 3 and 4).

**Table 3:** Demographic and clinical characteristics of the 77 PCP patients according to herpesvirus detection

	PCP without HV (n = 23)	PCP with at least one HV (n = 54)	*p* value
Mean age ± SD (years)	60.9 +/- 15.5	58.9 +/- 13.5	0.59^*^
Male sex, n (%)	14 (60.9)	36 (66.7)	0.90^**^
Underlying disease, n (%)			
Hematological malignancy	11 (47.8)	26 (48.1)	0.97^**^
Solid organ or hematopoietic stem cell transplant	11 (47.8)	15 (27.8)	0.08^**^
Solid tumor	5 (21.7)	9 (16.7)	0.83^**^
Autoimmune disease	1 (4.3)	8 (14.8)	0.35^**^
Lung disease	1 (4.3)	6 (11.1)	0.60^**^
HIV infection	0 (0)	5 (9.3)	0.31^**^
Comorbidity, n (%)			
Cardiovascular disease	10 (43.5)	22 (40.7)	0.82^**^
Diabetes	4 (17.4)	9 (16.6)	1^**^
Renal failure	4 (17.4)	5 (9.2)	0.52^**^
Immunosuppressive treatment, n (%)			
Corticosteroids	7 (30.4)	15 (27.7)	0.81^**^
Cytotoxic chemotherapy	8 (34.8)	15 (27.7)	0.53^**^
Antiviral treatment, n (%)	6 (26)	18 (33.3)	**0.04** ^**^

Bold font indicates statistical significance

HV, *Herpesviridae*; SD, standard deviation; PCP, *Pneumocystis pneumonia*

^*^Student’s test

^**^Chi-squared test

**Table 4:** Demographic and clinical characteristics of the 48 colonized patients according to herpesvirus detection

	Colonization without HV (n = 20)	Colonization with at least one HV (n = 28)	*p* value
Mean age ± SD (years)	62.2 +/- 12.6	63 +/- 13.1	0.84^*^
Male sex, n (%)	11 (55)	20 (71.4)	0.24^**^
Underlying disease, n (%)			
Hematologic malignancy	6 (30)	13 (46.4)	0.25^**^
Solid organ or hematopoietic stem cell transplant	2 (10)	11 (39.3)	0.05^**^
Solid tumor	5 (25)	2 (7.1)	0.20^**^
Autoimmune disease	4 (20)	1 (3.5)	0.17^**^
Lung pathology	8 (40)	10 (35.7)	0.76^**^
HIV infection	0 (0)	0 (0)	NA
Comorbidity, n (%)			
Cardiovascular disease	10 (50)	18 (64.2)	0.32^**^
Diabetes	0 (0)	6 (21.4)	0.07^**^
Renal insufficiency	0 (0)	2 (7.1)	0.62^**^
Immunosuppressive treatment, n (%)			
Corticosteroids	5 (25)	12 (42.9)	0.20^**^
Cytotoxic chemotherapy	4 (20)	8 (28.6)	0.49^**^
Antiviral treatment, n (%)	2 (10)	4 (14.3)	1^**^

HV, *Herpesviridae*; NA, not applicable; SD, standard deviation

^*^ Student’s test

^**^ Chi-squared test

**Fig. 2:**
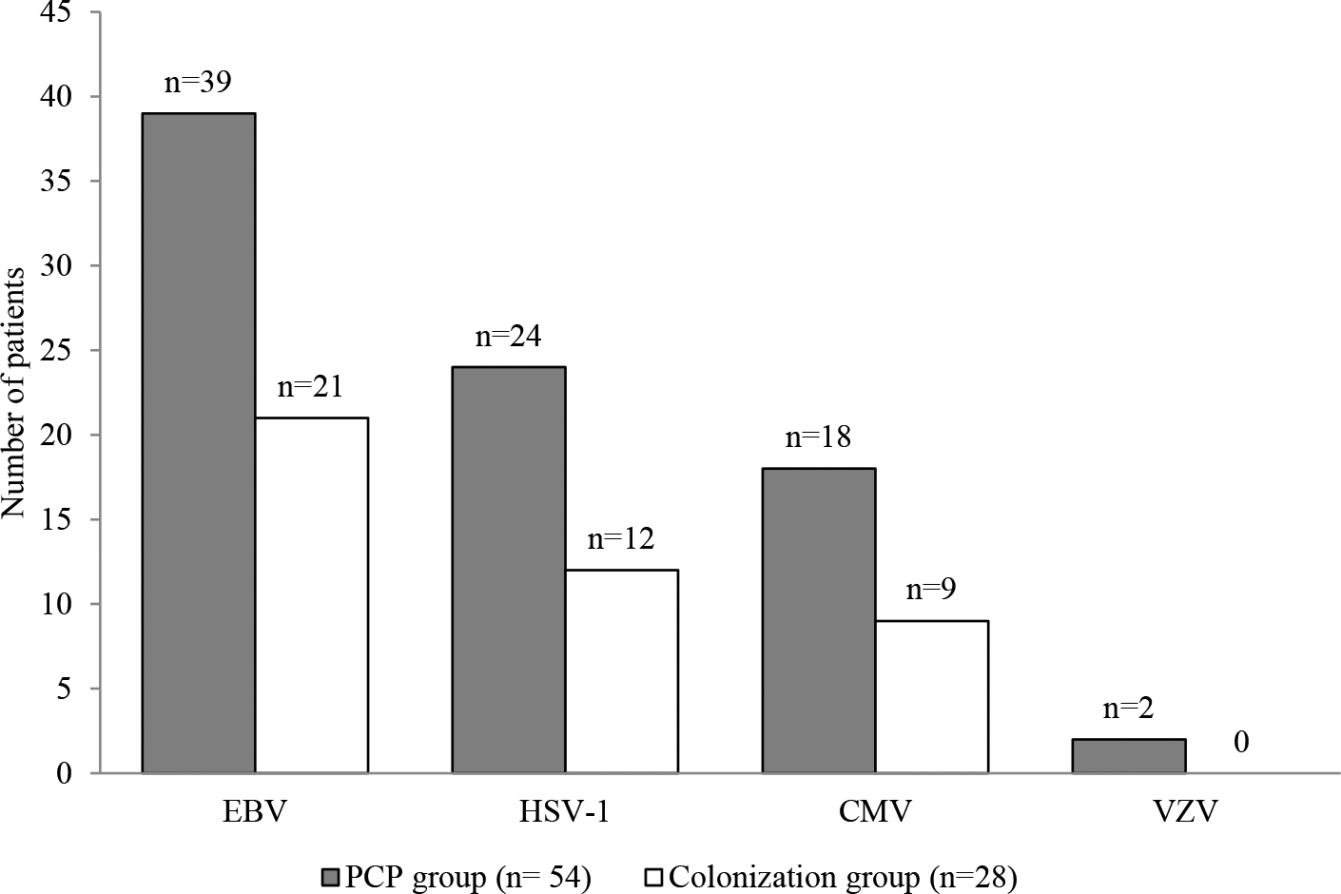
Patients with at least one *Herpesviridae* in bronchoalveolar lavage fluid sample according to *Pneumocystis* infection. CMV, Cytomegalovirus; EBV, Epstein-Barr virus; HSV1/2, Herpes simplex viruses type 1 and type 2; PCP, *Pneumocystis* pneumonia; VZV, Varicella zoster virus

**Fig. 3:**
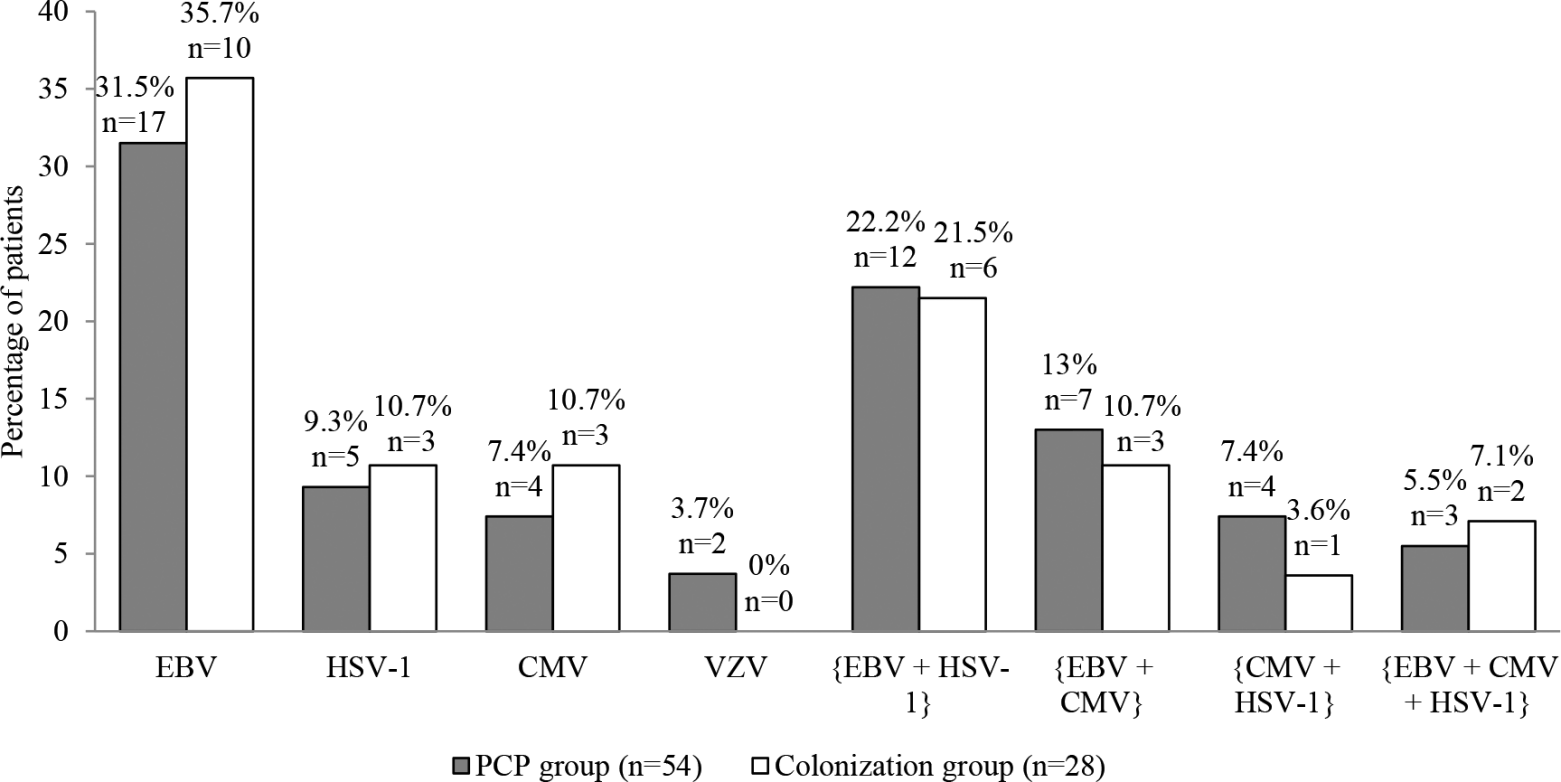
Patients with *Herpesviridae* detected alone or in combination according to *Pneumocystis* infection. CMV, Cytomegalovirus; EBV, Epstein-Barr virus; HSV1/2, Herpes simplex viruses type 1 and type 2; PCP, *Pneumocystis* pneumonia; VZV, Varicella zoster virus

Considering patients for whom at least one virus was detected, EBV, HSV-1, CMV, and VZV were observed in 39, 24, 18 and 2 of the 54 PCP patients and in 21, 12, 9 and 0 of the 28 colonized patients, respectively (Fig. 2). EBV, HSV-1, CMV, and VZV were detected alone in 17 (31.5%), 5 (9.3%), 4 (7.4%), and 2 (3.7%) PCP patients, and in 10 (35.7%), 3 (10.7%), 3 (10.7%) and 0 (0%) colonized patients, respectively. The following combinations [EBV + HSV-1], [EBV + CMV], [CMV + HSV-1] and [EBV + CMV + HSV-1] were detected in 12 (22.2%), 7 (13%), 4 (7.4%) and 3 (5.5%) PCP patients and in 6 (21.5%), 3 (10.7%), 1 (3.6%) and 2 (7.1%) colonized patients, respectively (Fig. 3). There was no significant difference in the distribution of HV between the two groups. HSV-2 was not detected in any sample.

### Survival analyses in the PCP group

**Fig. 4:**
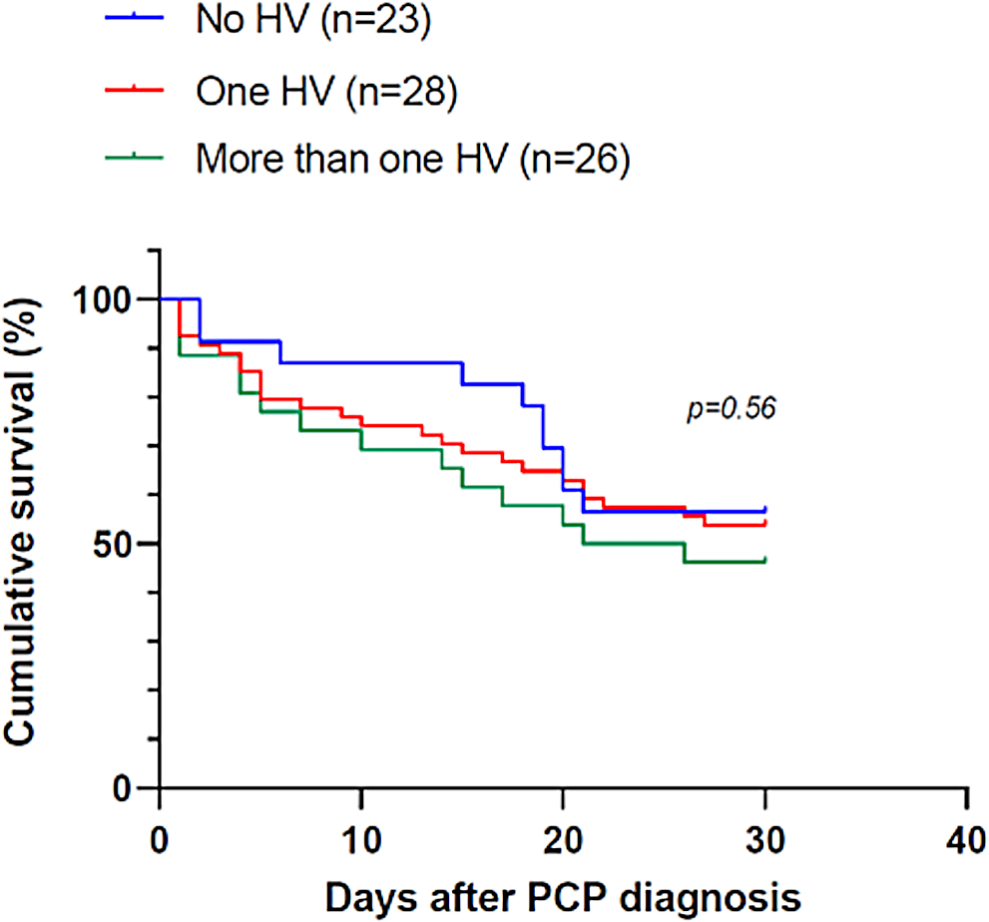
Survival analysis according to the result of *Herpesviridae* detection in the *Pneumocystis* pneumonia group. HV, *Herpesviridae*; PCP, *Pneumocystis* pneumonia

At day 30 after PCP diagnosis, the mortality rate was 43.5% (10/23), 39.3% (11/28) and 57.7% (15/26) in patients harboring no HV, one HV and more than one HV, respectively. No significant difference was observed (p = 0.56) (Fig. 4).

**Fig. 5:**
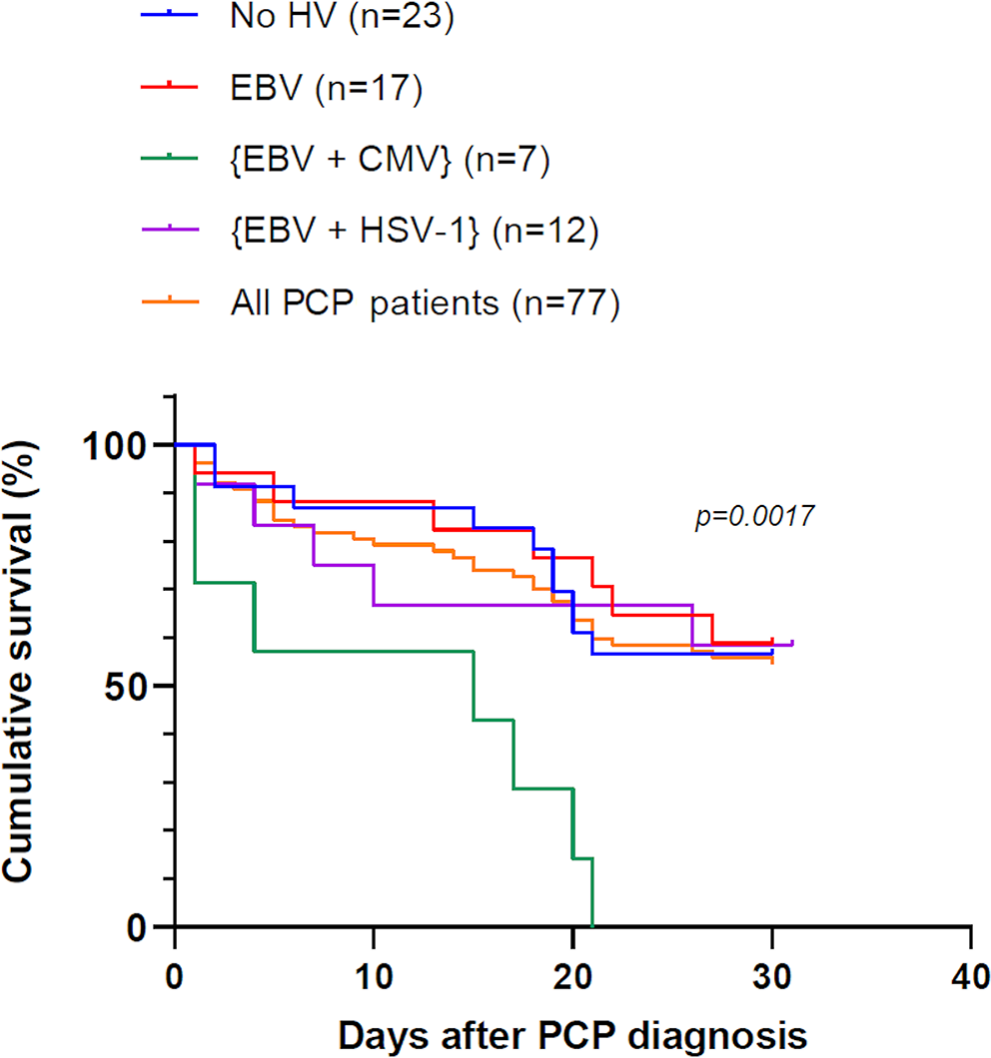
Survival analysis in *Pneumocystis* pneumonia patients according to different *Herpesviridae* patterns. CMV, Cytomegalovirus; EBV, Epstein-Barr virus; HSV-1, Herpes simplex viruses type 1; HV, *Herpesviridae*; PCP, *Pneumocystis* pneumonia

Nonetheless, a co-infection with [EBV + CMV] was significantly associated with an increased day-30 mortality rate in PCP patients compared to those without HV, with EBV alone and [EBV + HSV-1] (p = 0.0017) (Fig. 5). The other HVs associations could not be compared due to the low sample size. However, at day 30, among the 4 patients with CMV alone and the 5 patients with HSV-1 alone, none and 3 died, respectively.

In order to assess if increased risk of death was due to co-infection with [EBV + CMV] or if co-infection may be due to the fact that this group of patients were already more severely immuno-compromised than others, we analyzed the immunosuppressive treatment in PCP patients according to the distribution of different HVs. 28.6% of patients with [EBV + CMV] had received immunosuppressive treatments (corticosteroids and/or cytotoxic chemotherapy). This rate is comparable to that of patients with HSV, [EBV + HSV-1] and [EBV + CMV + HSV-1]. Furthermore, 50%, 75% and 76.4% of patients with [CMV + HSV-1], CMV and EBV had also received immunosuppressive treatment, respectively (Table 5). In other words, patients with [EBV + CMV] were not more severely immunocompromised than others.

**Table 5:** Immunosuppressive treatment (corticosteroids and/or cytotoxic chemotherapy) in the *Pneumocystis* pneumonia group with at least one *Herpesviridae* detected in bronchoalveolar lavage fluid sample

PCP patients with at least one *Herpesviridae* (n = 54)	Immunosuppressive treatment	
	**n**	**%**
EBV (n = 17)	13	76.4
HSV-1 (n = 5)	1	20
CMV (n = 4)	3	75
VZV (n = 2)	0	0
EBV + HSV-1 (n = 12)	4	33.3
EBV + CMV (n = 7)	2	28.6
CMV + HSV-1 (n = 4)	2	50
EBV + CMV + HSV-1 (n = 3)	1	33.3

CMV, Cytomegalovirus; EBV, Epstein-Barr virus; HSV1, Herpes simplex viruses type 1; PCP, *Pneumocystis* pneumonia; VZV, Varicella zoster virus

**Fig. 6:**
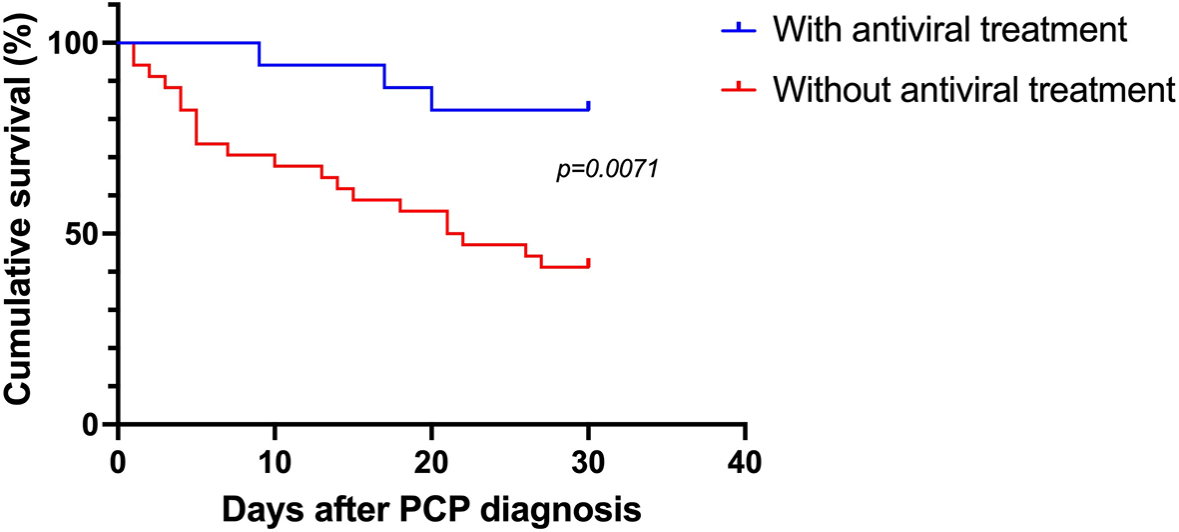
Survival analysis according to antiviral treatment in the *Pneumocystis* pneumonia group with at least one *Herpesviridae* detected in bronchoalveolar lavage fluid sample. PCP, *Pneumocystis* pneumonia

Moreover, among the 54 PCP patients with at least one *Herpesviridae* detected in bronchoalveolar lavage fluid sample, 18 were treated with antiviral drugs. Of these 18 patients, 4 (22%) died within 30 days after PCP diagnosis (mean: 11.5 days). Among the 36 patients who did not receive antiviral treatment, 22 (61%) died within 30 days after PCP diagnosis (mean: 10.2 days). The absence of antiviral treatment was significantly associated with an increased day-30 mortality rate in PCP patients with at least one *Herpesviridae* detected in BALF sample (Fig. 6, p = 0.0071).

## Disscussion

Although previous studies [8, 10, 11, 14, 17] have identified CMV or HSV co-infections as independent risk factors of mortality in HIV-negative patients with PCP, there are very few data concerning the presence and contribution of other HVs to PCP outcome. The present study provides supplemental data concerning the co-detection of *P. jirovecii* and HVs other than CMV and we observed that EBV was the most frequent virus in PCP patients. Moreover, to the best of our knowledge, the epidemiology of HVs in pulmonary colonization with *P. jirovecii* had never been investigated. CMV was detected in 9/48 (18.75%) colonized patients suggesting that the presence of CMV in the lungs of colonized patients is not a rare event, as previously described in PCP patients. Interestingly, in our study, EBV was the main virus in colonized patients group, followed by HSV-1 and CMV. Further studies are now warranted to confirm these observations and to assess their clinical significance. Here, we assessed the frequency of HVs, as well as their possible combinations, in BALF samples from PCP patients and colonized patients. Distribution of HVs between the two groups did not differ significantly, regardless of the type of HVs or their combination. This suggests that the presence of HVs was not related to the clinical form of *Pneumocystis* infection.

Besides, we compared results with a control group of *P. jirovecii-*negative patients in whom HVs detection was carried out over the same period (data not shown). Interestingly, HVs (alone or in combination) were significantly more frequent in *Pneumocystis*-infected group than in the non-infected group (p = 0.003). The role of HVs as respiratory pathogens is still unclear in immunocompromised patients infected with *Pneumocystis* but their presence should certainly not be overlooked since they could increase the adverse effects of the fungus in the pulmonary alveoli. In addition, it has recently been suggested that solid organ and HSC transplant patients presenting with CMV infection were at risk of developing invasive fungal diseases, such as aspergillosis or PCP [12, 17]. Neutropenia induced by antiviral treatment (ganciclovir) would especially increase the risk of developing co-infections [17].

There are conflicting reports regarding morbidity and mortality due to CMV co-infection during PCP. Some authors reported no significant difference of morbidity and mortality rates between PCP patients developing a simultaneous CMV pneumonia and those with no viral pneumonia [9, 20–23]. However, the presence of CMV in BALF samples from HIV-infected patients with PCP has been correlated with a poor prognosis [10, 24] and numerous studies described a relationship between CMV infection and increased mortality rate in critically ill and SOT patients who developed PCP [11, 18, 25–28]. In the present study, we assessed the relationship between different HVs and the mortality rate. Although the 30-day mortality did not vary significantly according to the result of HV detection, our data indicated that the combination of [EBV + CMV] was significantly associated with an increased 30-day mortality rate in PCP patients compared to those without HV, with EBV, [EBV + CMV] and [EBV + HSV-1] (p = 0.0017). This increased mortality appears to be independent from the deep of the underlying immunodeficiency and rather linked to the specific [EBV + CMV] co-infection. Moreover, the absence of antiviral treatment was significantly associated with an increased day-30 mortality rate in PCP patients with at least one *Herpesviridae* detected in BALF. These results suggest that HV infections are important to test and treat in patients with PCP.

The diagnosis of pulmonary mixed infections remains challenging but is crucial because clinical manifestations are severe and associate with poor prognosis [14]. Pulmonary infections associating *P. jirovecii* and more than one viruses has already been described [13, 19, 27, 29]. Maartens et al. conducted a prospective study including 284 HIV-infected patients with pulmonary symptoms [30]. Simultaneous testing for community-acquired bacteria and viruses, mycobacteria, CMV and *P. jirovecii* was performed using PCR assays in induced sputa from all patients. Various respiratory viruses were detected in 203/284 patients (71.5%) with the highest prevalence in PCP patients (22/26, 85%). CMV was detected in 5/26 patients with PCP (19.2%) and multiple co-infections with respiratory viruses such as Human metapneumovirus A/B, Enterovirus, Influenza A or Parainfluenza virus were identified in PCP patients. In our study, *Pneumocystis* and HVs detections were performed in either a single BALF sample or two BALF samples collected less than two days apart. Thus, we demonstrated the simultaneous presence of HVs and *P. jirovecii* in 70.1% of PCP patients and in 58.3% of colonized patients. Moreover, at least two HVs were detected in one-third of BALF samples, regardless of the *Pneumocystis* status. In this context, syndromic molecular testing targeting the main pulmonary microorganisms represents an interesting method to reduce time and cost related to pre-analytical process. The usefulness of syndromic approach has already been assessed with a multiplex PCR TaqMan^®^ array card (ThermoFisher) which simultaneously detects 24 viruses (including CMV and HSV 1 + 2), eight bacteria and two fungi (including *P. jirovecii*) [31] and with the “FTD respiratory pathogens 33” kit (Fast-Track Diagnostics, Esch-sur-Alzet, Luxembourg) targeting 33 microorganisms (including *P. jirovecii* and CMV) [30]. Recent studies proposed next-generation sequencing methods for a large-scale approach [13, 27, 29, 32]. These techniques allow thus prompt management of polymicrobial infections frequently associated with a poor prognosis.

## Conclusion

Our study describes the detection of HVs in the lungs from *Pneumocystis*-infected patients with either PCP or colonization over a 7-year period. Distribution of each HV, alone or in combination was similar whatever the clinical form of *Pneumocystis* infection and EBV was the most frequent virus. Multiple viral infections were also observed in one-third of patient from each group. In addition, our results suggest that an increased 30-day mortality rate can be observed in PCP patients, especially in the case of [EBV + CMV] co-infection and in the absence of antiviral treatment.

## Data Availability

All data generated or analysed during this study are included in this published article.
